# Anatomical similarity between the *Sost*‐knockout mouse and sclerosteosis in humans

**DOI:** 10.1002/ar.24318

**Published:** 2019-12-17

**Authors:** Uwe Y. Schwarze, Toni Dobsak, Reinhard Gruber, Fred L. Bookstein

**Affiliations:** ^1^ Department of Oral Biology, School of Dentistry Medical University of Vienna Vienna Austria; ^2^ Austrian Cluster for Tissue Regeneration Vienna Austria; ^3^ Department of Oral Surgery Medical University of Vienna Vienna Austria; ^4^ Department of Periodontology University of Bern Bern Switzerland; ^5^ Department of Anthropology University of Vienna Vienna Austria; ^6^ Department of Statistics University of Washington Seattle Washington

**Keywords:** sclerostin, mouse models, geometric morphometrics, cranial morphology

## Abstract

Sclerosteosis, a rare autosomal recessive genetic disorder caused by a mutation of the *Sost* gene, manifests in the facial skeleton by gigantism, facial distortion, mandibular prognathism, cranial nerve palsy, and, in extreme cases, compression of the medulla oblongata. Mice lacking sclerostin reflect some symptoms of sclerosteosis, but this is the first report of the effect on the facial skeleton. We used geometric morphometrics (GMM) to analyze the deformations of the murine facial skeleton from the wild‐type to the *Sost* gene knockout. Landmark coordinates were obtained by surface reconstructions from micro‐computed tomography. Centroid size, principal component scores in shape space and form space, and asymmetry were computed by the standard GMM formulas, and dental and skeletal jaw lengths were examined as ratios. We show here that, compared to wild type controls, mice lacking *Sost* have larger centroid size (effect size, *p*‐value: 4.59, <.001), higher mean asymmetry (1.14, .065), dental and skeletal mandibular prognathism (1.36, .010 and 5.92, <.001), a smaller foramen magnum (−1.71, .015), and calvaria that are more highly curved (form space *p* = 4.09, .002; shape space *p* = 12.82, .002). These features of mice lacking sclerostin largely correspond to the changes of the facial skeleton observed in sclerosteosis. This alignment further supports claims that the *Sost* gene plays a fundamental role in bony facial development in rodents and humans alike.

## 1 INTRODUCTION

The sclerostin protein, encoded by *Sost* (ten Dijke, Krause, de Gorter, Löwik, & van Bezooijen, 2008), inhibits the Wnt signaling pathway that helps prevent overgrowth of bone (van Bezooijen et al., 2007; Yavropoulou, Xygonakis, Lolou, Karadimou, & Yovos, 2014). Osteocytes are the major source of sclerostin (Chen et al., 2013; Papapoulos, 2011; Poole et al., 2005; ten Dijke et al., 2008; van Bezooijen, ten Dijke, Papapoulos, & Löwik, 2005), but cementocytes (Jäger, Götz, Lossdörfer, & Rath‐Deschner, 2010; Lehnen, Götz, Baxmann, & Jäger, 2012) and hypertrophic chondrocytes (van Bezooijen et al., 2009) also produce sclerostin. The function of sclerostin is indicated by sclerosteosis (MIM 269500) and Van Buchem Disease (hyperostosis corticalis generalisata; OMIM 239100; Hernandez, Whitty, John Wardale, & Henson, 2014; Kusu et al., 2003), both of which are genetic disorders of high bone mass. While the symptoms of Van Buchem Disease are milder, sclerosteosis, which is characterized by high bone density as well (Hernandez et al., 2014), manifests as severe malformations such as gigantism, facial bone deformations, asymmetric faces, prominence of the frontal bone, mandibular prognathism, and gross cranial hyperostosis with cranial nerve entrapment. Particularly severe cases are at risk of sudden death from compression of the brainstem by the foramen magnum (Balemans et al., 2001; Beighton, 1988; Hamersma, Gardner, & Beighton, 2003; Moester, Papapoulos, Löwik, & van Bezooijen, 2010; ten Dijke et al., 2008; Van Hul et al., 1998). This excessive bone building aspect leads to sclerostin antibody treatment in recent research (Asadipooya & Weinstock, 2019; Chavassieux et al., 2019; De Maré et al., 2019; Kleber, Ntanasis‐Stathopoulos, Dimopoulos, & Terpos, 2019; Korn et al., 2019).

Consistent with the sclerosteosis phenotype (Li et al., 2008), mouse models lacking *Sost* develop a high bone mass. The facial skeleton of *Sost‐*knockout (*Sost‐*KO) mice follows the expected pathological bone growth, but neither obvious facial distortions nor facial muscle paralysis have been reported (Li et al., 2008). Previously, Kuchler et al. (2014) described alveolar anatomy and tooth morphology in the *Sost*‐KO mouse, reporting that the ratio of alveolar nerve area to mandibular canal area is smaller in the knockouts. This prompted us to search for symptoms of sclerosteosis in the facial skeleton of this knockout model, asking whether the mouse model reflects the facial malformations observed in sclerosteosis to any useful degree.

The methodology we have chosen to exploit is geometric morphometrics (GMM), an exploratory toolkit for comparing morphologies within and between species. GMM is the statistical analysis of shape and size features, including pathologies, as they describe configurations of landmark points in two or three dimensions. Well established in anthropology (Weber & Bookstein, 2011) and paleobiology (Lawing & Polly, 2010), this method is increasingly being used in fields of medical research such as orthodontics (Bertl et al., 2016; Celar, Freudenthaler, Celar, Jonke, & Schneider, 1998), anatomy (Loth et al., 2015), psychology (Prasad et al., 2015), and craniofacial surgery (Segna et al., 2019; Trefný, Krajíček, & Velemínská, 2015). In earlier applications to mouse models, GMM has led to discoveries in ontogenetic development (Boughner et al., 2008), quantitative trait locus effects (Workman, Leamy, Routman, & Cheverud, 2002), relationships between gene and pathology (Green et al., 2015) and similarity of developmental processes to those of humans (Martínez‐Abadías et al., 2012).

The aim of the present study, an observational design, is to characterize the impact of the *Sost*‐KO on murine facial bone anatomy by examination of size, shape, asymmetry, jaw lengths, and the foramen magnum opening, all as characterized by GMM.

## 2 MATERIALS AND METHODS

### 2.1 Ethics statement

Protocols, handling, and care of the mice conformed to the Swiss federal law for animal protection under the control of the Basel‐Stadt Cantonal Veterinary Office, Switzerland. Sacrifice was done with CO_2_ to preserve the integrity of the skull.

### 2.2 Animals and micro‐computed tomography

Sclerostin knockout mice with a targeted disruption of the sclerostin coding region have been described previously (Li et al., 2008). To generate the *Sost*‐KO mice, first chimeric mice were created by using targeted ES cells derived from the 129/OlaHsd mouse substrain. Heterozygous *Sost*‐KO offspring of chimeric mice and C57BL/6 mice were backcrossed for four generations to C57BL/6 mice and then interbred to generate homozygous *Sost* mutant mice. All mice were kept in cages under standard laboratory conditions at a constant temperature of 25°C and a 12/12‐hr light–dark cycle. Mice received a standard rodent diet (3,302, Provimi Kliba SA, Switzerland) with water ad libitum (Kramer, Loots, Studer, Keller, & Kneissel, 2010).

**Figure 1:**
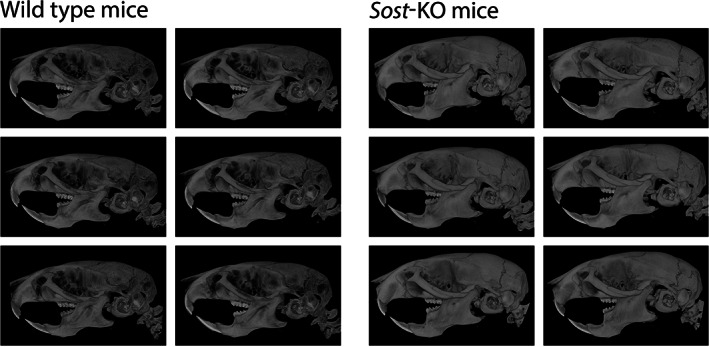
The mice skull sample used. Left side the six wild‐type mice; the right side the six *Sost*‐KO mice. The *Sost*‐KO mice are brighter indicating their higher bone density

We received the skulls of six female *Sost*‐KO (−/−) mice and six female wild‐type controls, *Sost* (+/+), 12 weeks of age at sacrifice, from Michaela Kneissel (Novartis Institutes for BioMedical Research, Musculoskeletal Disease Area, Basel, Switzerland), licensed from Deltagen, Inc. (San Mateo, CA; Figure 1). These are the same mice used in the study of Kuchler et al. (2014). The *Sost*‐KO (−/−) show higher bone mass and density than the WT mice (Video 1: https://players.brightcove.net/656326989001/default_default/index.html?videoId=6115072461001). Heterozygote (*Sost* (+/−)) mice were not analyzed because heterozygote humans are clinically normal (Beighton, 1988) and mild differences would be hard to detect.

Microcomputed tomography (μCT) at a resolution of 20.5 μm was carried out using a vivaCT75 (SCANCO Medical AG, Brüttisellen, Switzerland). The scanning of the skulls was done at 70 kV/114 μA with an integration time of 300 ms (Kuchler et al., 2014).

### 2.3 Data acquisition

**Figure 2:**
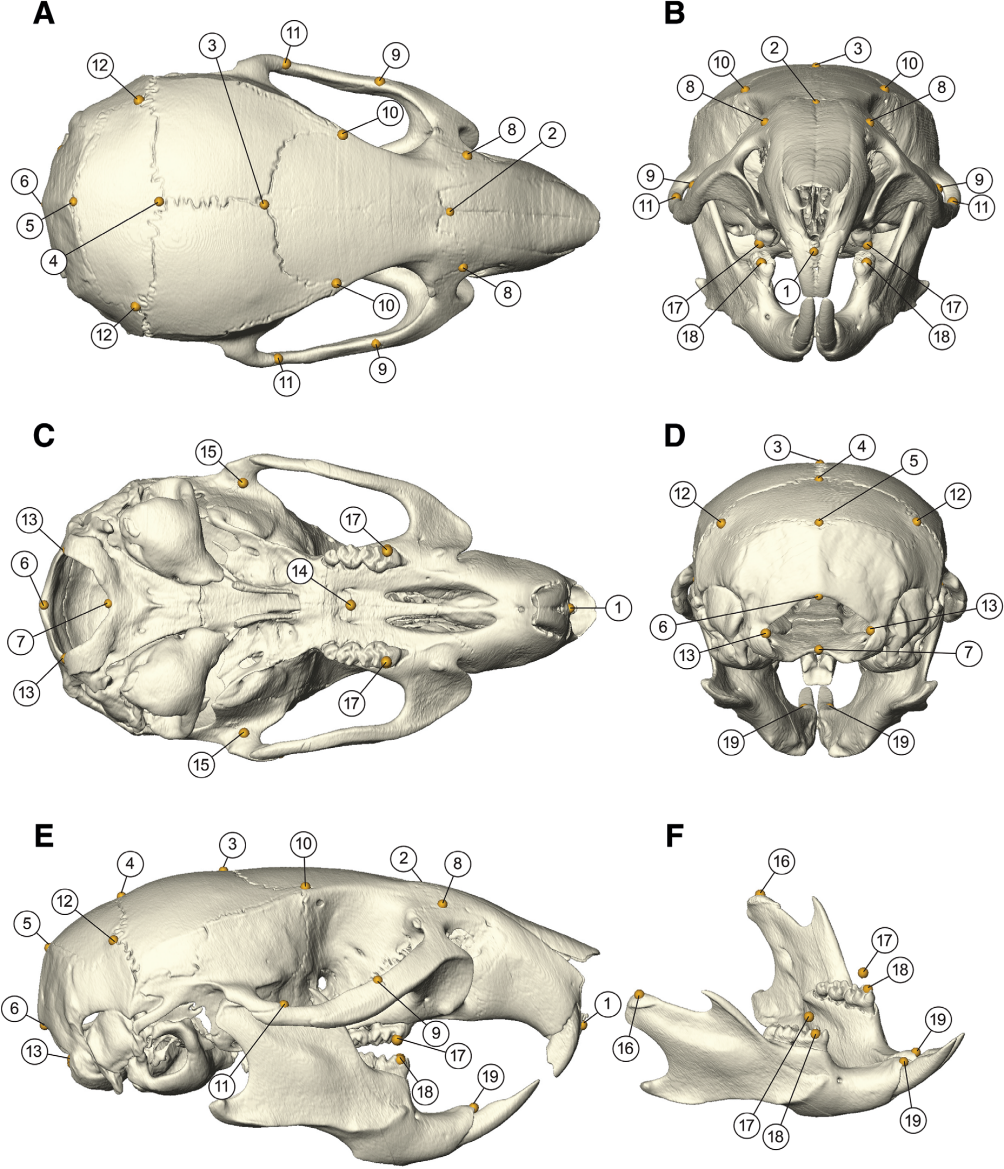
Landmark configuration on a *Sost*‐KO surface reconstruction. We established a landmark set on defined points, definitions see Table 1, with eight midline landmarks (LM) and 11 bilateral landmarks were located on each surface. centroid size calculation, principal component analysis, uniform component, and partial warp 1 calculations of the skull uses seven midline landmarks (LM:1–7) and six bilateral landmarks (LM: 8–13). Landmarks 14–19 are used with previous mentioned for distance measurements such as length, height, and jaw lengths

Bony surfaces of these skulls were generated by Amira (Version 5.6, Visage Imaging Inc., San Diego, CA) using the half maximum height value as described by Weber and Bookstein (2011). We established a landmark set of 30 defined points: Eight midline landmarks (LM) and 11 bilateral landmarks on each surface (Figure 2, Table 1). Centroid size calculation, principal component analysis (PCA), and uniform component and partial warp 1 calculations of the skull use seven midline landmarks (LM: 1–7) and six bilateral landmarks (LM: 8–13).

**Table 1:** Definition of landmarks

No.	Landmarks definition	Used in
1	Most anterior point in the median sagittal plane on the alveolar process between the incisors.	CS, L/H, PCA, MiCS, SL
2	Intersection of nasofrontal suture and the median sagittal plane.	CS, PCA, MiCS
3	Intersection of the sagittal suture and the coronal suture.	CS, PCA
4	Intersection of sagittal suture and anterior lamdoid suture.	CS, PCA
5	Intersection of the posterior lambdoid suture and median sagittal plane.	CS, PCA, OCS
6	Intersection of vertex of the posterior margin of the foramen magnum and the median sagittal plane.	CS, L/H, PCA
7	Intersection of vertex of the anterior margin of the foramen magnum and the median sagittal plane.	CS, L/H, PCA
8. l. r.	Intersection of maxillopraemaxillary suture, praemaxillofrontal suture and maxillofrontal suture.	CS, PCA
9. l. r.	Most lateral part on superior margin of the maxillary zygomatic suture.	CS, PCA
10. l. r.	Most anterior point of the coronal suture.	CS, PCA
11. l. r.	Upper midst of the temporozygomatic suture in superior aspect.	CS, PCA
12. l. r.	Intersection of anterior lambdoid suture, posterior lambdoid suture and the lambdoid suture.	CS, PCA, OCS
13. l. r.	Most lateral point of vertex of the margin of the foramen magnum.	CS, PCA
14	Intersection of transversa palatina sutura and median palatine suture	L/H, MiCS
15. l. r.	Midpoint of the mandibular fossa in basal aspect.	DL, SL
16. l. r.	Midpoint of the mandible caput in superior aspect.	MaCS, DL, SL
17. l. r.	Mesio‐buccal cusp of the upper first molar.	DL
18. l. r.	Sulcus between the mesial and distal buccal cusp of the lower first molar.	DL
19. l. r.	Midpoint of the lingual side of the alveolar margin of the incisors.	MaCS, SL

Abbreviations: CS, centroid size; DL, dental length; L/H, length to height ratio; MiCS, midface centroid size; MaCS, mandible centroid size; PCA, principal component analysis; OCS, occipital centroid size; SL, skeletal length.

Gigantism is measured by calculating the centroid size (sum of squares of all the landmarks and the average of them all). This measurement is the standard for assessing size in geometric morphometric analysis in general.

Facial deformation is investigated by exploiting three morphometric analyses: PCA, which emphasizes large scale differences in shape; partial warp analysis, used here to capture the group differences in the ratio of length to height and in longitudinal curvature; and asymmetry analysis, which assesses shape differences between left and right hemicrania. All three are standard tools of today's GMM; for a recent textbook review, see Bookstein (2018, Chapter 5).

Skull asymmetry, the third way to quantify the facial deformation, induced by lack of sclerostin was determined by calculating the total asymmetry consisting of directional and fluctuating asymmetry. Total asymmetry, the Procrustes distance between any form and its reflected relabeling, was used as an additional quantification of perturbed development *via* the formulas for total asymmetry (and associated significance tests) from Mardia, Bookstein, and Moreton (2000).

Distances between specific landmarks were calculated in accord with the symptoms of sclerosteosis, see also Table 1. The length to height ratio was calculated by dividing the distance of landmark 1–6 as length by the distance from landmark 3 and to the average of LM 14 and 7 as height.

Mandibular prognathism as described by Angle (1899) is diagnosed with a closed mouth and occluding teeth. The positions of the mesio‐buccal cusp of the upper first molar and the sulcus between the mesial and distal buccal cusp of the lower first molar are then evaluated. This method is not feasible in dead mice without large errors. To be analogous to how mandibular prognathism is diagnosed in dentistry, we measured the distance between the temporomandibular joint (TMJ; upper jaw LM: 15l, 15r lower jaw LM: 16l, 16r) and the described points of angle (upper molar LM: 17l, 17r; lower molar LM: 18l, 18r). We refer to these distances as upper and lower dental jaw distance.

Mandibular prognathism in the sense of a visibly protruding mandible includes additionally the parts up to the chin. To assess this, distances between the above‐mentioned TMJ landmarks and the furthest definable points on the jaws were used (upper jaw LM: 1, lower jaw LM: 19l, 19r). We refer to these distances as upper and lower skeletal jaw distance.

A ratio of lower jaw distance to upper jaw distance was calculated. To exclude the possibility of a hypoplastic midface we calculated the ratios of centroid size of the occipital part of the individual skull (LM: 4, 12l, 12r) to centroid size of the midface (LM: 1, 2, 14) and the mandible (LM: 16l, 16 r, mean of 19l and 19r), respectively.

Constriction of nerves secondary to hyperostosis is proposed to have the underlying pathological mechanism of increased bone formation, by osteoblasts (Van Hul et al., 1998), and therefore, we assume, relative smaller bony openings such as canals or foramina. The size of the major foramen is quantified by measuring the vertical diameter (LM: 6, 7) and a horizontal diameter (LM: 13l, 13r), then correcting the ratio to centroid size.

### 2.4 Statistical analysis

We transferred the coordinates of the landmarks into a text file in morphologika format for use in the EVAN Toolbox 1.71 (EVAN‐Society e.V., Vienna, Austria, 2014). A generalized Procrustes analysis was performed to superimpose the landmark configurations, quantify centroid size, and calculate the Procrustes distances and Procrustes shape coordinates that are conventionally used to display dimensions of maximum variation of shape or of form (shape‐and‐size) pooled across the groups (see in general Bookstein, 2018).

As graphic representations of morphological differences, we constructed thin plate splines and relative warp visualizations using extreme scores on the first PC in shape space.

For significance tests, we used Mann–Whitney tests. Tests and graphics were performed using GraphPad Prism version 7.04 for Windows (GraphPad Software, La Jolla, CA, www.graphpad.com).

**Table 2:** Summary of statistical values by measurements

	Minimum		Average		Maximum		Standard deviation		Effect size (*p*‐value)
Measurement\mouse	WT	*Sost*‐KO	WT	*Sost*‐KO	WT	*Sost*‐KO	WT	*Sost*‐KO	
CS size (mm)	32.077	33.372	32.525	33.913	32.789	34.382	0.252	0.346	4.59 (.001)
Length/height	3.128	2.944	3.183	3.036	3.231	3.147	0.041	0.069	−2.6 (.004)
Principal component 1 in shape space	−0.025	0.000	−0.018	0.018	−0.013	0.036	0.004	0.012	4.09 (.002)
Principal component 1 in form space	−0.036	0.025	−0.028	0.028	−0.023	0.035	0.005	0.003	12.82 (.002)
Ratio midface/CC	0.592	0.576	0.607	0.595	0.612	0.626	0.008	0.019	−0.85 (.132)
Ratio mandible/CC	0.806	0.829	0.829	0.859	0.840	0.874	0.012	0.017	2.04 (.026)
Total individual asymmetry	0.006	0.004	0.009	0.014	0.012	0.020	0.002	0.006	1.14 (.065)
Lower/upper jaw dental	1.033	1.042	1.045	1.068	1.068	1.101	0.012	0.021	1.36 (.010)
Lower/upper jaw skeletal	0.748	0.778	0.755	0.788	0.761	0.798	0.005	0.007	5.92 (<.001)
FM vertical (mm)	3.747	3.627	3.838	3.701	3.924	3.815	0.068	0.066	−2.05 (.009)
FM horizontal (mm)	7.556	7.972	7.892	8.332	8.092	8.577	0.195	0.233	2.05 (.009)
Ratio FM vertical/CS	0.115	0.107	0.118	0.109	0.120	0.112	0.002	0.002	−4.52 (.002)
Ratio FM horizontal/CS	0.236	0.236	0.243	0.246	0.248	0.257	0.005	0.008	0.47 (.394)
Major foramen (vertical diameter/CS) × (horizontal diameter/CS)	0.027	0.025	0.028	0.027	0.029	0.028	0.001	0.001	−1.71 (.015)

## 3 RESULTS

Inferential statistics are summarized in Table 2: Minima, means, maxima, standard deviation, effect size, and *p* values, are given. A summary of distance measurements can be found in Table 3.

**Table 3:** Summary of distance measurements

	Mean		Standard deviation		Effect size
Mouse	WT	*Sost*‐KO	WT	*Sost*‐KO	
FM vertical (mm)	3.84	3.70	0.07	0.07	−2.05
FM horizontal (mm)	7.89	8.33	0.20	0.23	2.05
Dental lower jaw l, r mean (mm)	7.29	7.83	0.10	0.17	3.75
Dental upper jaw l, r mean (mm)	6.98	7.33	0.16	0.15	2.21
Skeletal lower jaw l, r mean (mm)	10.97	11.84	0.10	0.11	8.08
Skeletal upper jaw l, r mean (mm)	14.53	15.02	0.19	0.25	2.20
CS of occipital (mm)	13.07	13.41	0.22	0.24	1.47
CS of midface (mm)	7.94	7.98	0.08	0.19	0.27
CS of mandible (mm)	10.83	11.51	0.07	0.08	9.09
Length (mm)	21.73	22.30	0.17	0.41	1.83
Height (mm)	6.83	7.35	0.10	0.06	6.48

Note: These are measurements between landmarks that are used for ratios or length and the ratios itself by individual mice. Including the mean of the specific groups.

### 3.1 Size

**Figure 3:**
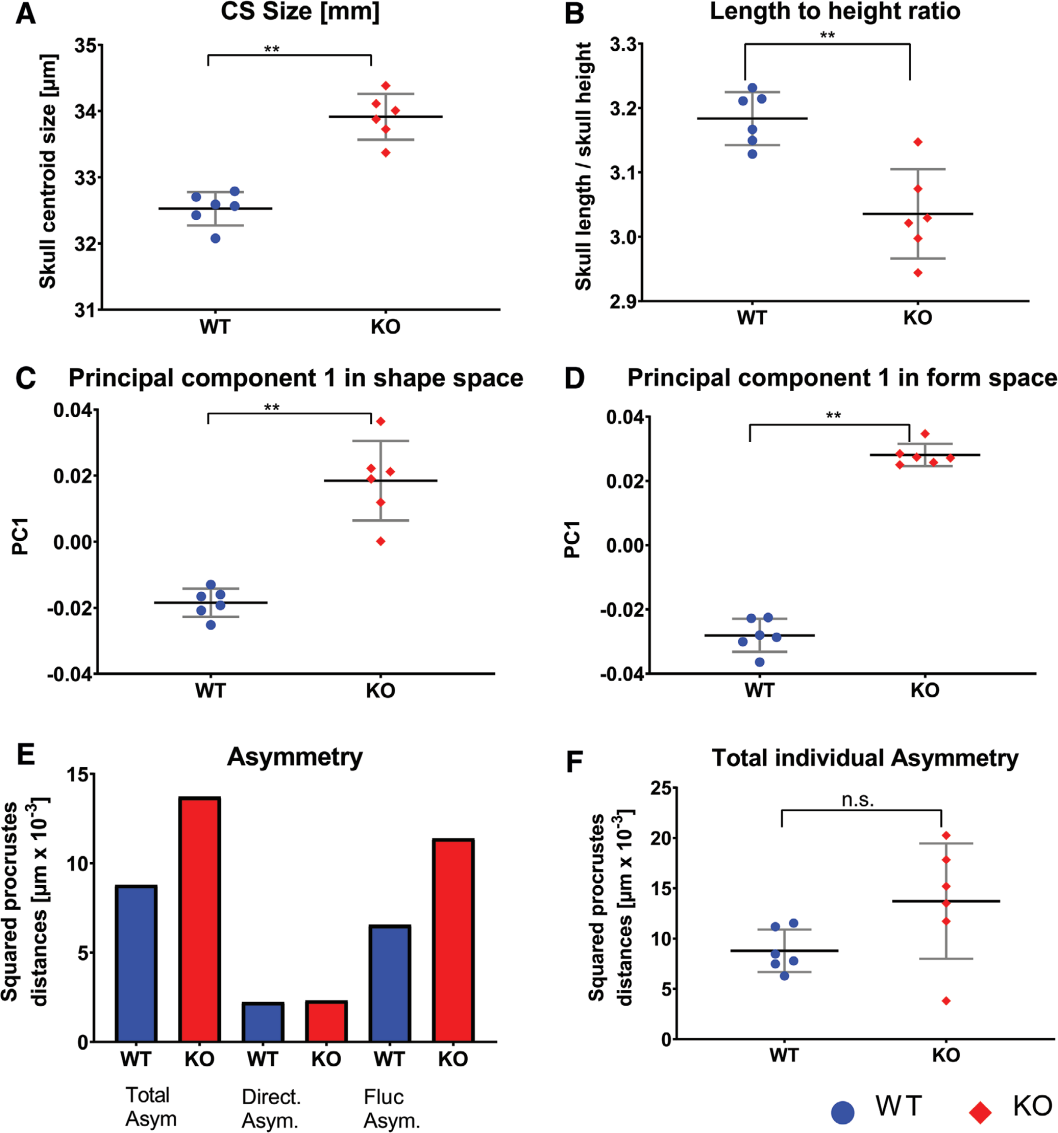
Graphic representation of statistical evaluation. (A) Scatterplots of centroid size, (B) length to height ratio, (C) principal component in shape and (D) form space, (E) separated asymmetry, and (F) total asymmetry by individual. Asterisks indicate significant differences and n.s. stands for not significant

Sizes of the six animals each in the WT and *Sost‐*KO groups were compared using the preferred GMM scalar, centroid size (Figure 3A). The average centroid size of the WT group was 32.5 mm versus 33.9 mm for the *Sost‐*KO group, with standard deviations of 0.25 and 0.35, respectively. There is complete separation of the groups in size (effect size: 4.59 *p*‐value: .0005; Figure 3A).

### 3.2 Shape and form analysis

**Figure 4:**
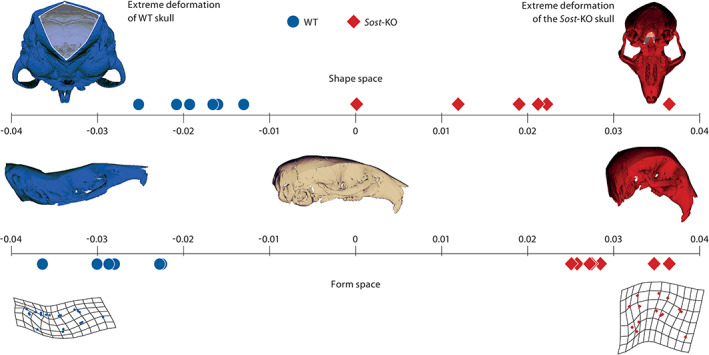
Deformation of the mouse skull according to principal component one in shape space (above) and in form space (below). The ivory skull in the center is the average skull form. Either blue skull on the left is an extreme (15‐fold) deformation of this mean form in the WT direction; the corresponding red skull represents the opposite deformation. In both GMM spaces, the groups are perfectly separated, the lower *Sost*‐KO skull showing higher curvature and a greater height‐to‐length ratio. The upper skulls show the deformation of the foramen magnum, which is reduced in diameter in the *Sost*‐KO group. These match aspects of skull deformations described in sclerosteosis

**Figure 5:**
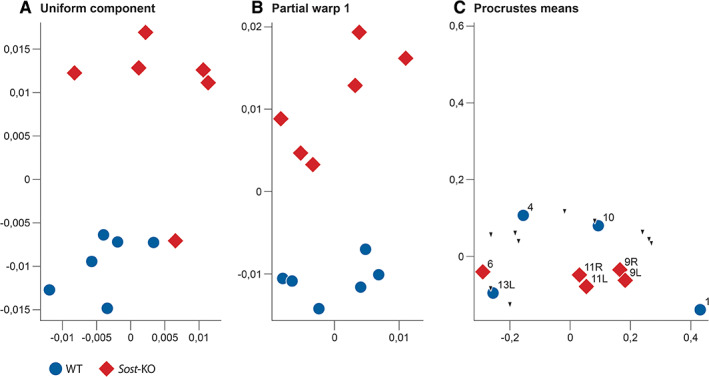
Graphic representation of uniform shape changes and grouping of landmarks of *Sost*‐KO mouse no. 2. The uniform is the part of the deformation where parallel lines remain parallel and the partial warp one is the uniform shape change. Uniform component and partial warp one give indication about differences in the length–height ratio. Since *Sost*‐KO mouse number two is an outlier regarding the uniform component, a Procrustes means plot for the skull configurations with the highlighted grouping of *Sost*‐KO mouse number two was needed. This plot indicates the landmarks of *Sost*‐KO mouse number two that are located with the respective group. The Procrustes means plot landmark that contribute to the curving are either neutral or grouped with the WT group, bilateral landmarks that group with the WT group are one sided 10L and 13L so this seems to be a part of the asymmetry. Grouped with the *Sost* group are the landmarks number 6, 9L, R and 11L, R. Landmark 6 belongs to the major foramen and probably indicates the smaller opening. The other two bilateral landmarks belong to the zygomatic arc where they may represent robustness features

To find shape differences we carried out two standard PCAs of these configurations, one in shape space (excluding size) and one in form space (including size). In both, the first PC perfectly separates our two groups (effect size: shape space 4.09; form space 12.82; Figures 3C,D and 4) We end our report with this first PC because in both spaces the second PC was not significant according to the decision procedure of Bookstein (2014; figure 5.12). The shape difference can be divided into two different general factors: First, a curvature term, the *Sost‐*KO group has a more convex calvaria then the WT controls as seen as bending lines in the grids of Figure 4, lowest row; second, a stretching term, the height to length ratio is smaller in the *Sost‐*KO group than in the WT‐group. The grids in Figure 4 indicate this by rectangles that are taller than they are wide in the *Sost‐*KO group but wider than they are tall in the WT‐group. The length to height ratio in Figure 3B is significantly different (effect size: −2.6) with a mean length to height ratio of 3.183 for the WT group and 3.036 for the *Sost‐*KO group. The Uniform component (Figure 5A) separates the groups except for one *Sost‐*KO Mouse (#2) that is located with WT mice. Partial warp again separates the groups perfectly (Figure 5B). The Procrustes means for the skull configurations with the highlighted grouping of *Sost‐*KO Mouse #2 (Figure 5C) indicate the landmarks of that mouse that are located with the respective group. *Sost‐*KO mouse #2 landmarks located in the WT group are 1, 4, 10l, and 13l; those located in the *Sost‐*KO group are 6, 9l, 9r, 11l, and 11r; the other landmarks are located indifferent to the groups. *Sost*‐KO mouse #2 is different she is the largest mouse by centroid size, yet closest to the WT group in shape space on PC1 (Figure 4), and groups more with the WT in the uniform component plot (Figure 5A). The Procrustes means plot (Figure 5C) shows that landmarks that contribute to the curving are either group‐indifferent or grouped with the WT group. Paired landmarks that group with the WT group are one‐sided 10L and 13L so this seems to be an expression of asymmetry. Grouped with the *Sost*‐group are landmarks 6, 9L, R and 11L, R. Landmark 6 lies on the foramen magnum and so probably indicates that its opening is smaller (see below). The other two bilateral landmarks belong to the zygomatic arch, where they may represent robustness features.

### 3.3 Asymmetry analysis

Skull asymmetry due to lack of sclerostin was assessed *via* total asymmetry. The values calculated and presented are group values, since directional and fluctuating asymmetry depend on the squared Procrustes distance between the original mean and the reflected mean of the landmarks. Total asymmetry is increased in the *Sost*‐KO group by 56%, compared to the WT group (Figure 3E). The analysis of total asymmetry by individuals (Figure 3F) shows no significant difference between the groups (effect size: 1.14). However, the standard deviation is higher in *Sost‐*KO group compared to the WT (WT: 0.002, KO: 0.006). Consequently, mice with *Sost*‐KO have higher means total asymmetry than the WT controls (without quite reaching the level of significance).

### 3.4 Jaw length

**Figure 6:**
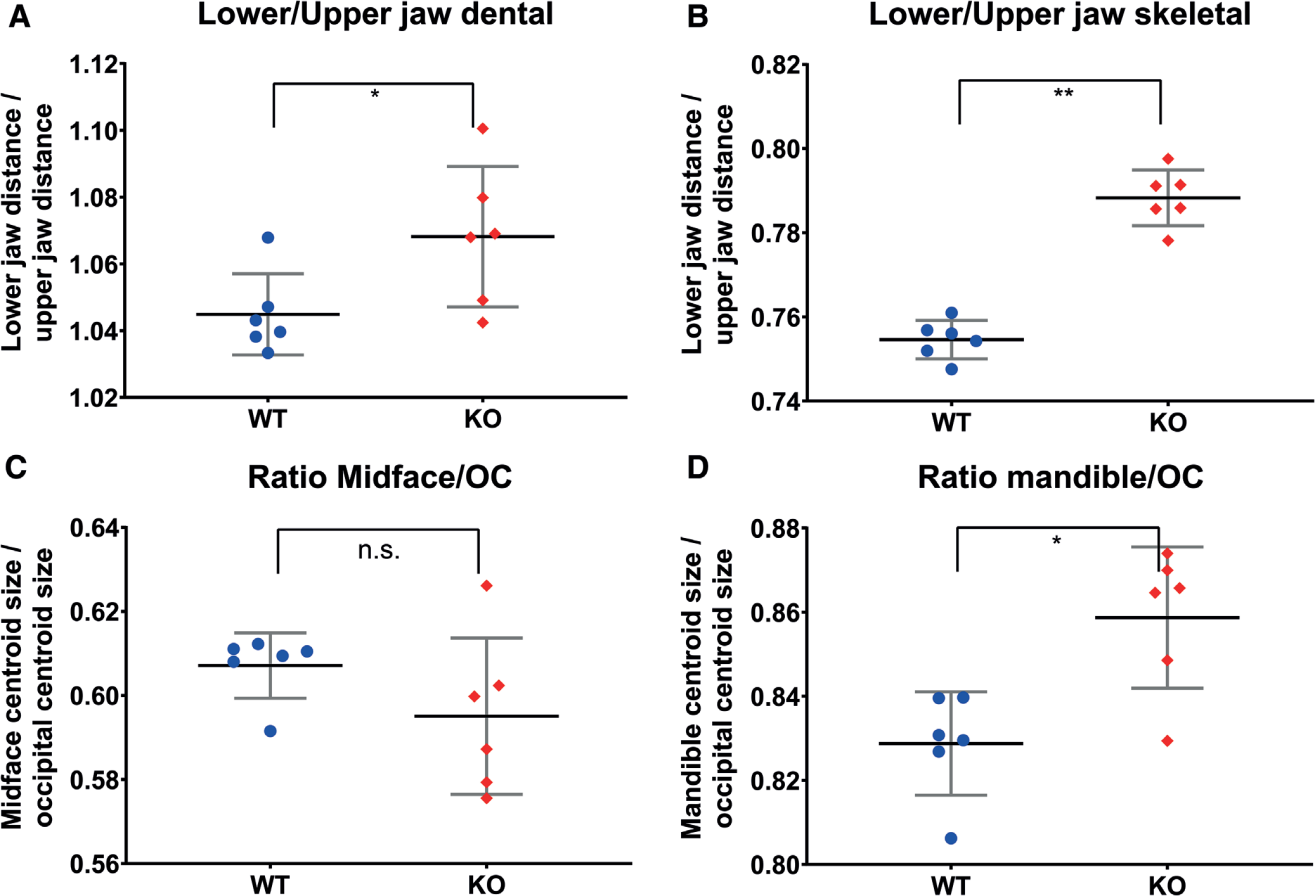
Graphic representation of statistical evaluation of the jaws. Scatterplots of dental and skeletal ratio of jaw length, midface, and mandible size ratio with the occipital part centroid size as standard. Asterisk indicates significant differences and ns stands for not significant. In dental and skeletal ratios, the mandible is relatively larger to exclude a hypoplasia midface size of the midface and mandible are standardized with taking the ratio by the centroid size of the occipital part. A mandibular prognathism is indeed present in *Sost*‐KO mice taken together the measurements

Jaw length findings are displayed in Figure 6A,B. The dental and skeletal ratios are significantly higher in the Sost‐KO group than in the WT group (effect size: dental: 1.36; skeletal: 5.92 this indicates that the mandible is larger than the upper jaw. The ratio of the centroid size of the midface and mandible divided by the centroid size of the occipital part (OC, occipital centroid size) shows no significant difference in midface OC ratio (Figure 6C) between the groups (effect size: −0.85; Mean WT: 0.607, KO: 0.595). The mandible OC ratio (Figure 6D) is larger in the *Sost*‐KO group than the WT group (effect size: 2.04; Mean WT: 0.828, KO: 0.859)—there is indeed a mandibular prognathism in the mice similar to the symptom described for sclerosteosis patients. Dental ratio, skeletal ratio, midface size ratio, and mandibular size ratio all are higher in the *Sost‐*KO group than the WT group.

### 3.5 Foramen magnum diameter

**Figure 7:**
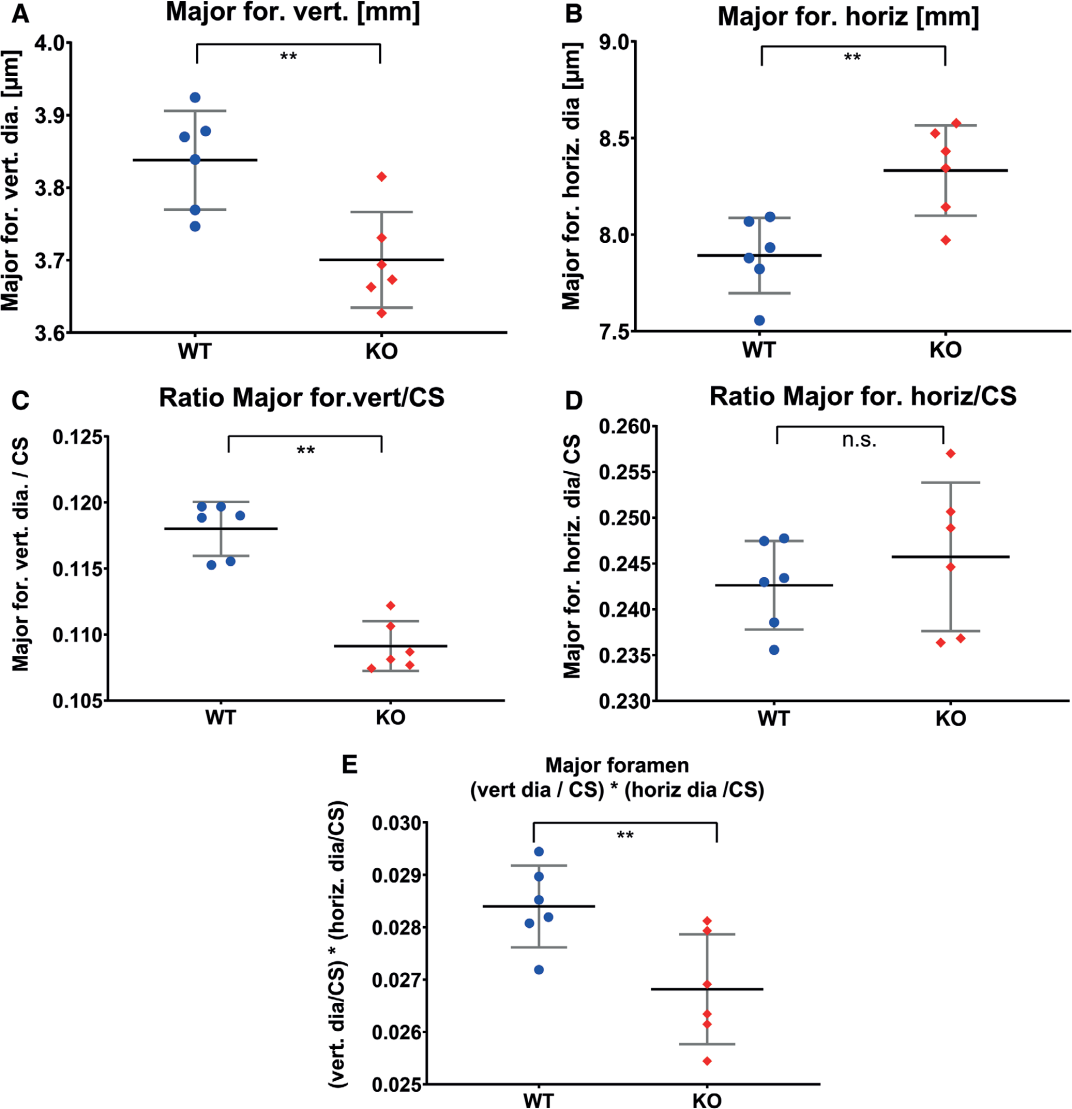
Graphic representation of statistical evaluation of foramen magnum. Scatterplots of horizontal and vertical diameter in absolute number and relative ratios with the centroid size as standard. Asterisk indicates significant differences and ns stands for not significant. The lack of sclerostin impact differs on the horizontal and vertical diameter. The horizontal diameter seems to be less affected than the vertical. Together the foramen magnum opening is relatively significant smaller in the *Sost*‐KO group

The foramen magnum morphology is depicted in the relative warps of PC1 in shape and form space in Figure 4 top, skulls after deformations corresponding to about 15 times the extremes of the WT and *Sost‐*KO tendencies. The measured horizontal and vertical diameters in scatterplots Figure 7A–E indicate an absolutely and relatively smaller vertical diameter of the Foramen magnum (Fig. 7A,C) in the *Sost‐*KO group (effect size: absolutely: −2.05; relatively: −4.52). The horizontal diameter is absolutely larger (effect size: 2.05) in the *Sost‐*KO groups but not relatively (effect size: 0.47). The product of both diameters, a surrogate for foramen magnum area, is significantly smaller for the *Sost*‐Ko group compared to the WT (effect size: −1.71).

## 4 DISCUSSION

The *Sost‐*KO mouse model mimics the condition of human sclerosteosis more accurately than previous investigations have realized (Li et al., 2008). The mice skulls lacking sclerostin (a) are larger in size than their littermates, (b) have deformations of the skull represented as more highly curved calvaria, and (c) have higher mean asymmetry although the difference is not statistically significant. Moreover, *Sost‐*KO mice showed (d) dental and skeletal mandibular prognathism and also (e) a relatively smaller foramen magnum opening. We might imagine three potential causes of such a phenotype: Gene defects leading to developmental stress, higher bone mass *per se*, or the changes in biomechanical stress consequent on the higher bone mass. We review these in order.

Any gene defect that causes developmental stress or uncanalized development results in the inability of an organism to compensate (canalize) a stress, in this case, the absence of a protein (sclerostin; Schaefer, Lauc, Mitteroecker, Gunz, & Bookstein, 2006; Wilkins, 1997). This developmental stress is often found associated with excess morphological variance or higher asymmetry (Schaefer et al., 2006). Consistent with this explanation, the *Sost‐*KO mouse group shows higher asymmetry. Asymmetry is associated with the ability of organisms to express the genotype as a three‐dimensional phenotype (Palmer & Strobeck, 1986); failure to produce a perfect symmetric skull is manifested at the level of bone tissue by different apposition rates and different fusion times of sutures in the developing organism. With a higher bone mass, there seems to be a higher probability of failure in the symmetric apposition of bone and the fusing of sutures at different time points. Although asymmetry has not yet been quantified in human sclerosteosis, the phenotypic expression is often obvious and asymmetrical distortions of the facial skeleton have indeed been reported (Beighton, 1988; Hamersma et al., 2003; Moester et al., 2010).

Excess of variance is another consequence of gene defects that are connected with dysregulation of development and homeostasis (Palmer & Strobeck, 1986). Such an excess of variance was apparent in *Sost‐*KO mice in regard to size, position in shape space, length to height ratio, asymmetry by individual, jaw length ratios, centroid size ratios, and horizontal diameter of the foramen magnum. We suggest that future studies of human sclerosteosis explicitly consider variances of the facial skeleton in the same way.

Higher bone mass is characteristic of both *Sost‐*KO mice and human sclerosteosis (Beighton, 1988; Hamersma et al., 2003; Li et al., 2008; Moester et al., 2010). We have confirmed this global finding while adding some geometric details for the mice that may have human clinical analogues. The larger size, smaller diameter of the foramen magnum, and longer mandibles are characteristic of anabolic bone remodeling when the Wnt antagonist is missing (Beighton, 1988; Hamersma et al., 2003; Moester et al., 2010). The smaller nerve opening is also histologically confirmed by our previous work on the dental and periodontal phenotype of *Sost*‐KO mice the infra‐alveolar canal is relatively smaller. Bone overgrowth includes increased size and consequent diminished free space of foramina and canals, the consequences of which for their contents can be severe. We conjecture that differential biomechanical loading causes the imbalanced growth of the upper and lower jaws in both species. Our hypothesis is that the protruding of the mandible is part of a “vicious circle” where bone apposition on the mandible leads to higher mandible mass, which leads to higher masticatory muscle forces, which leads to higher mechanical stress, which leads to higher bone mass. In addition, this circle leads in consequence to a larger mandible, which protrudes.

Differences in biomechanical loading on the skull may be direct or may be secondary to the higher bone masses we have reported (Li et al., 2008; Wolff, 1892). Following Wolff's law, this hypothesis is consistent with the observation by Lightfoot and German (1998) that mice with muscular dystrophy have flatter and more elongated skulls. In human sclerosteosis as well, the heavier skull results in larger muscle forces and thus higher mechanical strains on the human skull that might induce deformations such as protrusion of the frontal bone and the mandible (Beighton, 1988). A mouse skull and a human skull work differently. A protrusion of the frontal bone, in the sense of growing more anterior, as it is seen in humans is not probable. However, a bone more highly curved to resist stronger mechanical stress is exactly what we see in the mice and what we have described and is homologous to the probable mechanics in humans.

In all these ways, our findings support the use of *Sost‐*KO mice as a model for human sclerosteosis. The *Sost*‐KO mouse two incorporates features of both groups but still can be allocated to the *Sost*‐KO group in shape space and is within the *Sost*‐KO group in the form space. The reason for this can only be hypothesized. One reason can be that size could play a role she is the largest mouse, so the expression rate of certain features at the larger size would have less effect on the curvature of the skull than on the zygomatic arch, which is of muscle origin. Another hypothesis would be that there are individual causes that led to that phenotype.

Our claims must be accompanied by some limitations. Mainly, the comparisons between mice and men remain descriptive, as no human quantitative data are available. However, this is common practice in medicine. Thus, the clinical relevance of the present findings for sclerosteosis patients remains a matter of speculation at this time. Heterozygotic mice were not analyzed since the signal is probably much weaker and would need a higher sample size. Although the sample size with six mice in each group can be considered small, the phenotypic difference is strong, produces significant different results and in accordance with the three Rs in animal welfare, we have used as few animals as possible; to use more would not be beneficial for the study, thus morally problematic. Taken together, our findings argue strongly for a morphometric component in future studies in humans and in sclerosteosis patients, and argue as well for the potential importance of studying long‐term effects of sclerostin antibody treatment in the mouse model, specifically, its effects on malformations, mandibular prognathism, and nerve entrapment. Two kinds of further studies are suggested: Morphometric quantification of the deformations observed in humans, and investigations of the therapeutic potential of sclerostin antibody treatment in experimental mice.

## 5 CONCLUSION

The impact of the *Sost*‐KO on murine facial bone anatomy includes larger size, large differences in overall anterior–posterior curvature and length‐to‐height ratio, higher mean asymmetry, mandibular prognathism, and relatively smaller nerve opening. In all these ways, *Sost*‐KO mice mimic anatomical features of human sclerosteosis. These findings suggest two kinds of further studies: Quantification of the deformations observed in humans, and similar investigations of the therapeutic potential of sclerostin antibody treatment in experimental mice.

## CONFLICT OF INTEREST

The authors declare no potential conflicts of interest with respect to the authorship and/or publication of this article.

## AUTHOR CONTRIBUTIONS

U.S., R.G., and F.B. designed the study. U.S. performed the study. U.S. and T.D. collected data. U.S. and F.B. analyzed the data. U.S., T.D., R.G., and F.B. collected the interpretation of data. U.S., R.G., and F.B. drafted the manuscript. U.S., T.D., R.G., and F.B. revised the manuscript content. U.S., T.D., R.G., and F.B. approving the final version of manuscript. U.S. and F.B. takes responsibility for the integrity of the data analysis.

## Supporting information


**Video S1** Rotation and then horizontal slices through a WT and a *Sost*‐KO mouse. The mous skull to the left is a WT mouse the Skull to the right is a *Sost*‐KO skull. Virtual slices start at landmark 3 and 4 goes horizontally through the skull until the lower end of the mandible. Higher density is indicated by brighter gray values, *Sost*‐KO to the right shows higher bone mass.

**Figure S1** A μCT superimposition of a wild type skull in blue to green and a SOST KO skull in red to yellow, made in Amira, is pictured. With geometric morphometrics these two skulls with the most morphological differences are found, using a principal component analysis. The symptoms of sclerosteosis, a rare disease with bone overgrowth, for which the SOST KO mouse is a model, can be seen in this superimposition. Symptoms that can be seen in this visualization. Gigantism is visualized by the fact that the red SOST KO skull surrounds the smaller blue WT skull. Distortion of facial bones are assessed by the non‐parallel differences of the bone tissue and the high asymmetry marked by the appearance of blue WT parts such as the left zygomatic arc. Mandibular prognathism is clearly visible in the lateral view by comparing the relation between upper and lower jaw. Nerve entrapment by smaller diameter of bony openings is difficult to see in this visualization because of the gigantism, but the foramen magnum is relatively smaller in SOST KO than in WT. Therefore this superimposition displays the characteristics in SOST KO mice that were revealed by geometric morphometrics and can be related to sclerosteosis in humans.
