# Supplementary figures and images for: Anatomical similarity between the Sost‐knockout mouse and sclerosteosis in humans
Source: Anat Rec (Hoboken). 2019 Dec 17;303(9):2295–308. doi: 10.1002/ar.24318 (PMC7496997; doi:10.1002/ar.24318)

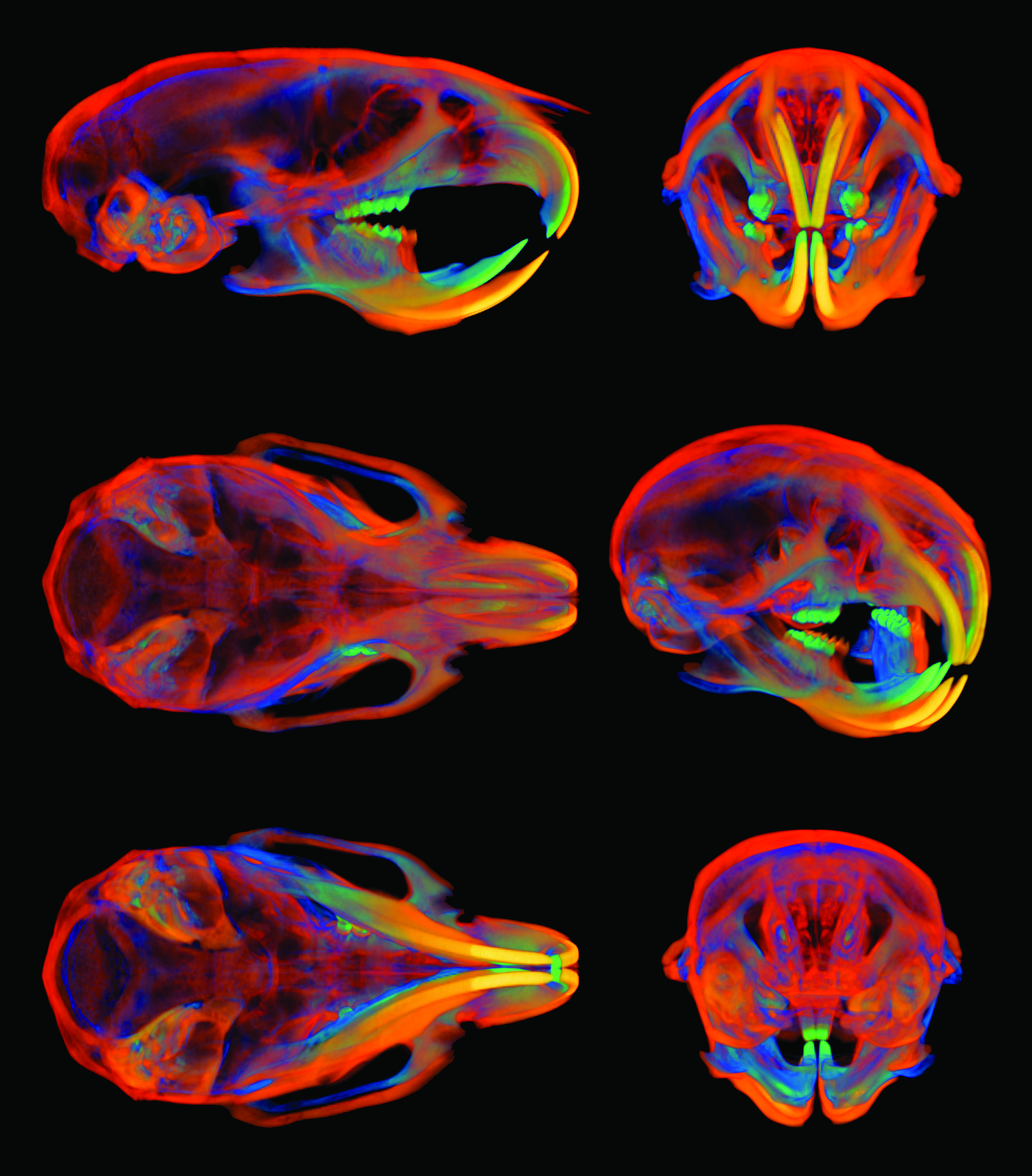

Supplement: Supplementary file 2 — Figure S1 A μCT superimposition of a wild type skull in blue to green and a SOST KO skull in red to yellow, made in Amira, is pictured. With geometric morphometrics these two skulls with the most morphological differences are found, using a principal component analysis. The symptoms of sclerosteosis, a rare disease with bone overgrowth, for which the SOST KO mouse is a model, can be seen in this superimposition. Symptoms that can be seen in this visualization. Gigantism is visualized by the fact that the red SOST KO skull surrounds the smaller blue WT skull. Distortion of facial bones are assessed by the non‐parallel differences of the bone tissue and the high asymmetry marked by the appearance of blue WT parts such as the left zygomatic arc. Mandibular prognathism is clearly visible in the lateral view by comparing the relation between upper and lower jaw. Nerve entrapment by smaller diameter of bony openings is difficult to see in this visualization because of the gigantism, but the foramen magnum is relatively smaller in SOST KO than in WT. Therefore this superimposition displays the characteristics in SOST KO mice that were revealed by geometric morphometrics and can be related to sclerosteosis in humans. [file AR-303-2295-s002.tif]
